# Bariatric surgery in a patient with cystinuria 

**DOI:** 10.5414/CNCS110496

**Published:** 2021-05-07

**Authors:** Melissa R. Nemati, Peter C. Harris, Andrea G. Cogal, David S. Goldfarb

**Affiliations:** 1 New York University, New York, NY,; 2 Division of Nephrology and Hypertension, Mayo Clinic, Rochester, MN,; 3 Nephrology Division, NY Harbor VA Medical Center, and NYU School of Medicine, New York, NY, USA

**Keywords:** autosomal recessive, cystinuria, bariatric surgery, gastric sleeve, genetic disease, kidney/abnormalities, kidney calculi, nephrocalcinosis

## Abstract

We recently encountered concern about the safety of bariatric surgery for a patient with cystinuria. Bariatric surgery procedures include those that cause malabsorption, like the Roux-en-Y gastric bypass procedure, and restrictive operations, such as the sleeve gastrectomy. These procedures produce beneficial effects on health and life expectancy, though whether kidney stones are prevented, as well as promoted, is not established. Although the importance of body weight to metabolic stone activity in patients with cystinuria is not established, the patient’s physicians were concerned about whether any bariatric surgery procedure would affect her ability to drink sufficient quantities of water in order to reduce stone activity. Here we report the experience of a genetically defined patient with cystinuria who underwent a gastric sleeve procedure. In the months after the procedure, she lost 45 kg, though with time she regained 23 kg of that loss. She was able to maintain a urine volume of 4.0 L per day and has had no stone recurrence.

## Introduction and background 

Kidney stones are increasing in prevalence in the United States [1]. A variety of potential causes has been implicated, including the role of overweight and weight gain. Increasing body mass index is associated with the metabolic syndrome, and the number of features of the metabolic syndrome correlates with kidney stone prevalence [2]. These associations are clear for uric acid stones and probably relate to the prevalence of calcium stones as well, but have not been investigated with respect to cystinuria. As overweight increases in prevalence, rates of bariatric surgery directed at weight loss increase as well [3]. Such surgery can have beneficial effects on health, though whether kidney stones are prevented, as well as promoted, is not yet established [4]. Bariatric surgery may entail procedures which promote malabsorption, such as the Roux-en-Y gastric bypass operation, or restrictive procedures, exemplified by laparoscopic banding and sleeve gastrectomy [5]. The former, associated with enteric hyperoxaluria, may be particularly associated with increased incidence of calcium oxalate stone formation, nephrocalcinosis, and chronic kidney disease [4]. We recently encountered concern about the safety of bariatric surgery for a patient with cystinuria. Although the importance of body weight to metabolic stone activity in patients with cystinuria is not established, her physicians were concerned about whether the surgery would affect her ability to drink sufficient quantities of water in order to reduce stone activity. Here we report the experience of a patient with cystinuria who underwent a gastric sleeve procedure. 

## Case 

A 48-year-old woman with cystinuria presented to the kidney stone prevention clinic to ask whether she could undergo bariatric surgery. The patient read this case report and gave us permission to submit it for publication. Her relevant medical history began with a first episode of renal colic with a stone at the age of 42 years; the stone passed without complication and was not recovered. Three years later, she underwent extracorporeal shockwave lithotripsy directed at a large stone in the left kidney, which was found to be composed of cystine. Because some residual non-obstructing stones were noted after the lithotripsy, she underwent ureteroscopy as well. She was treated with tiopronin and potassium citrate. She saw a nephrologist who continued her treatment with potassium citrate but switched her thiol therapy to D-penicillamine, thinking that, despite the short course, the tiopronin had not been effective. He advised her to have a water intake of 4 L per day and to reduce salt and animal protein intake. 

The family history was significant for calcium stones in her younger sister, who had had a parathyroidectomy. No other family members had a history of cystinuria, related genitourinary, or metabolic problems. Her medication doses included D-penicillamine 500 mg 3 times a day and potassium citrate 30 meq twice a day. She also took lamotrigine for depression, zolpidem, clonazepam, cranberry extract, levothyroxine, vitamin B6, vitamin B12, and vitamin D. 

On physical exam, the patient was 104.3 kg and 155.4 cm tall with a body mass index (BMI) of 43.2 kg/m^2^, meeting the definition of obesity. Blood pressure was 148/84 mmHg and there was no edema at the time of the examination. Physical examination was otherwise unremarkable. 

Serum chemistry test results were unremarkable, with creatinine concentration of 0.7 mg/dL, potassium 3.9 meq/L, bicarbonate 20 meq/dL, uric acid 6.9 mg/dL, and calcium 9.0 mg/dL. Liver function tests were normal and the glycosylated hemoglobin was 5.8%. Table 1 demonstrates the results of 24-hour urine collections. Two collections had been done at the time of her visit; the table demonstrates the mean result of the two collections. Cystine excretion was 990 mg per day after the ureteroscopy; on subsequent collections, she had 470 mg and then 746 mg 2 months prior to initial consultation. On the initial readings, the protein catabolic rate (PCR) was at the high end of the normal range at 1.1 gm/kg/d, but went down to 0.8. 

She reported a long history of attempted weight loss, depression, and significant unhappiness with her body image. She had been told that any type of bariatric surgery was not advised given her stone disease, particularly the requirement to maintain high urine volume. She was adherent to an appropriate diet but asked to increase her dosage of potassium citrate in order to reduce her fluid intake, which she found to be “exhausting”. 

We were impressed with her resolve regarding the option of bariatric surgery and apparent adherence with the medical regimen prescribed. We suggested that the patient consider gastric banding as a possible alternative to the Roux-en-Y gastric bypass procedure, as it would be more easily reversed if stone formation were to become overwhelming. 

After review of our consult note, the bariatric surgery team agreed to install a non-adjustable gastric sleeve, and performed the procedure 7 months later without complication. At follow-up 6 months after the procedure, the patient reported a weight loss of 45 kg and a significant improvement in her mood, affect, and performance. She continued to maintain a low sodium, near-vegetarian diet. She had stopped the vitamin D and the clonazepam. 

On physical examination, blood pressure was 118/79 mmHg, pulse was 70 beats per minute, and she had no pedal edema. Ultrasound revealed a 3 mm echo-density located in the right kidney with no definite shadow. A non-contrast computed tomography scan (CT) of the abdomen done 11 months later, 17 months after the surgery, showed no stones. 

It was concluded that the bariatric surgery was a success with no definite evidence of active stone disease. At the time of this report, nearly 6 years after the bariatric surgery, she was maintaining her weight at 68 kg, more than 50 kg below her peak, with BMI of 28.3 kg/m^2^. She has not had a recurrent symptomatic stone. The result of the post-operative 24-hour urine collection, performed 17 months after the procedure, is given in Table 1. She had a high urine volume, less sodium and cystine excretion with a relatively alkaline urine. Five years after the procedure, she had normal serum electrolytes: creatinine concentration of 0.6 mg/dL, sodium 138 meq/L, potassium 4.0 meq/L, chloride 99 meq/L, bicarbonate 26 meq/dl, uric acid 5.8 mg/dl, and calcium 9.4 mg/dL. 

The patient’s history was of a relatively mild nature, given the range of stone burden experienced by patients with cystinuria. We therefore determined the patient’s genotype. A previously described nonsense mutation was detected, in heterozygosity, in *SLC3A1*: c.1400T>C (p.M467T). Further analysis of her DNA was performed by testing with a specific Cystinuria Multiplex Ligation-dependent Probe Amplification (MLPA) panel (MRC-Holland, Amsterdam, The Netherlands) looking specifically for large deletions or duplications within the two cystinuria genes, *SLC3A1* and *SLC7A9*. The result was demonstrative of a previously described large duplication in *SLC3A1* consisting of exon 5 through exon 9. In summary, her cystinuria was not atypical, in that she had biallelic mutations affecting *SLC3A1*. 

## Discussion and literature review 

There are no previously published papers regarding bariatric surgery in cystinuria, a kidney stone disorder for which fluid intake is critical. The benefits of bariatric surgery for overweight people are significant, as it results in effective weight loss with associated improvement in metabolic syndrome, resolution of diabetes mellitus, and lowering of blood pressure [6]. These medical benefits are accompanied by improvements in affect and body image, as experienced by our patient. 

However, bariatric surgery also is associated with a notable risk of kidney stones and reduced glomerular filtration rate (GFR) [7]. This risk is, in part, attributed to the development of hyperoxaluria, which is not likely to be significant in cystinuria. Enteric hyperoxaluria has frequently been demonstrated after the Roux-en-Y gastric bypass, attributed to fat malabsorption. Steatorrhea is then associated with binding of calcium to fat in the intestinal lumen, leaving oxalate free to be absorbed. In addition, malabsorption of bile salts leads to increased colonic oxalate absorption [8]. Hyperoxaluria is associated with stones, and also nephrocalcinosis, a cause of reduced GFR. As reversal of the Roux-en-Y gastric bypass is considered a daunting surgical procedure, the development of significant kidney stones and chronic kidney disease is considered ominous. These changes in colonic oxalate absorption have not been seen with the gastric sleeve. Increases in urinary oxalate excretion have not been noted after gastric sleeve restrictive surgery [9]. 

One study did suggest that cystine is capable of reducing the solubility of calcium oxalate in vitro [10]. In another study, several patients with calcium stones, who underwent genotyping in search of potential monogenic causes of stones, were shown to be heterozygotic for mutations in *SLC3A1* or *SLC7A9*. Whether these mutations were simply incidental findings, versus somehow causative mutations, was not known. Of interest, in a recent study we found that ~ 32% of *SLC3A1* mutations were large rearrangements only detected by MLPA, suggesting that some apparent monoallelic patients may be biallelic if all possible mutations are accounted for [11]. Whether patients with cystinuria, or heterozygous carriers of mutated genes for cystinuria have increased risk for stones of non-cystine composition, is not known, but other potential metabolic risk factors have been demonstrated [12]. Since cystine is more soluble in alkaline urine, many patients with symptomatic stones receive potassium citrate to achieve urinary alkalinization [13]. Patients treated with potassium citrate could theoretically form more calcium phosphate stones due to urinary alkalinization [14], but this would not be expected to be associated with or exacerbated by enteric hyperoxaluria. 

Other mechanisms by which bariatric surgery could worsen or cause underlying stone disease, including cystine stones, are worth considering. Patients who have undergone bariatric surgery may experience a reduction of urine volume of ~ 0.5 L, an effect that is attributed to earlier satiety reducing fluid intake [15]. The result would be an increase in the supersaturation of any poorly soluble solute. To what extent stone-forming patients can overcome this effect by forcing fluid intake despite satiety has not been studied. A lower urine pH could result from losses of potential base in the stool post intestinal bypass, which could worsen uric acid or cystine stone formation; a reduction in urine citrate would also occur from increased stool losses of alkali-equivalents, which would contribute to exacerbation of calcium (either oxalate or phosphate) precipitation, but should not have an effect on cystine solubility per se [16]. 

We discussed these risks with our patient. She was motivated to lose weight and desired a procedure as a solution to a psychologically debilitating condition which she had struggled to overcome. She seemed very adherent to fluids and citrate therapy. Her urine volume was high, her urine calcium excretion low and she had never had a calcium stone. She understood that worsening of her stone disease was possible, but that fluid intake and alkali could also prevent such an exacerbation [17]. While the Roux-en-Y procedure may have been most effective for weight loss and associated with the best long-term outcomes, we recommended the gastric sleeve as a compromise: as a less extreme and more reversible option. The weight loss she achieved represented an outstanding result for which she was quite satisfied. She has not had recurrent stones. We judged the ultrasound depiction of a 3 mm stone as a false positive finding, given the subsequent negative CT scan. We do not think that bariatric surgery is absolutely contraindicated in patients with a history of kidney stones, though we note that this patient’s kidney stone history was relatively mild, in comparison to that of many patients with cystinuria. Our conclusion could very well be different for patients with calcium stones, with or without hyperoxaluria, in whom risk for worsening stone burden, nephrocalcinosis, and chronic kidney disease may increase [4]. Long-term registries of patients undergoing bariatric surgery that include kidney stones of varying composition, estimates of GFR, and measurement of urine chemistries, are warranted. 

## Statement of ethics 

The patient read this case report and gave us permission to submit it for publication. 

## Funding 

This work was supported by The Rare Kidney Stone Consortium (RKSC) part of the Rare Diseases Clinical Research Network (RDCRN), an initiative of the Office of Rare Diseases Research (ORDR), the National Center for Advancing Translational Sciences (NCATS), and the National Institute of Diabetes and Digestive and Kidney Diseases (NIDDK). The consortium is funded through collaboration between NCATS and the NIDDK (U54DK083908-01). We appreciate the continued support of the International Cystinuria Foundation, which has consistently encouraged our work. The RDCRN, NCATS, and NIDDK were not involved in the preparation of this manuscript. 

## Conflict of interest 

Nemati: none; Harris: none; Cogal: none; Goldfarb: received honoraria for lectures and served as a consultant for Retrophin/Travere; owner, Dr. Arnies, Inc.; funding from NIDDK, NCATS. 

**Table 1.** Results of 24-hour urine collections. The “pre-op” values are the means of two collections done before she underwent a bariatric procedure. The “post-op” values are derived from a single collection performed 17 months after the bariatric procedure.

	Pre-op 24-h urine	Post-op 24-h urine	Normal range
Volume (L/d)	3.4	4.2	0.5 – 4
Cr24 (mg/d)	1,491	1,663	15 – 20 mg/kg
Ca24 (mg/d)	85	97	< 250
Cit24 (mg/d)	1,013	941	> 450
Cys24 (mg/d)	990	470	< 75
Sodium (meq/d)	147	91	< 100
pH	7.14	7.18	5.8 – 6.2
PCR (gm/kg/d)	1.1	0.8	0.8 – 1.4
